# Factors Associated with the Time of Admission among Notified Dengue Fever Cases in Region VIII Philippines from 2008 to 2014

**DOI:** 10.1371/journal.pntd.0005050

**Published:** 2016-10-25

**Authors:** Jason Echavez Abello, Julita Gil Cuesta, Boyd Roderick Cerro, Debarati Guha-Sapir

**Affiliations:** 1 Universite catholique de Louvain, Center for Research on the Epidemiology of Disasters, Brussels, Belgium; 2 Department of Health, Regional Epidemiology and Surveillance Unit VIII, Tacloban City, Philippines; 3 Universite catholique de Louvain, Center for Research on the Epidemiology of Disasters, Brussels, Belgium; Australian National University, AUSTRALIA

## Abstract

In cases of Dengue fever, late hospital admission can lead to treatment delay and even death. In order to improve early disease notification and management, it is essential to investigate the factors affecting the time of admission of Dengue cases. This study determined the factors associated with the time of admission among notified Dengue cases. The study covered the period between 2008 and 2014 in Region VIII, Philippines. The factors assessed were age, sex, hospital sector, hospital level, disease severity based on the 1997 WHO Dengue classification, and period of admission (distinguishing between the 2010 Dengue epidemic and non-epidemic time). We analysed secondary data from the surveillance of notified Dengue cases. We calculated the association through chi-square test, ordinal logistic regression and linear regression at p value < 0.05. The study included 16,357 admitted Dengue cases. The reported cases included a majority of children (70.09%), mild cases of the disease (64.00%), patients from the public sector (69.82%), and non-tertiary hospitals (62.76%). Only 1.40% of cases had a laboratory confirmation. The epidemic period in 2010 comprised 48.68% of all the admitted cases during this period. Late admission was more likely among adults than children (p<0.05). The severe type of the disease was more likely to be admitted late than the mild type (p<0.05). Late admission was also more likely in public hospitals than in private hospitals (p<0.05); and within tertiary level hospitals than non-tertiary hospitals (p<0.05). Late admission was more likely during the non-epidemic period than the 2010 epidemic period (p<0.05). A case fatality rate of 1 or greater was significantly associated with children, severe diseases, tertiary hospitals and public hospitals when admitted late (p<0.05). Data suggests that early admission among child cases was common in Region VIII. This behavior is encouraging, and should be continued. However, further study is needed on the late admission among tertiary, public hospitals and non-epidemic period with reference to the quality of care, patient volume, out of pocket expense, and accessibility We recommend the consistent use of the 2009 WHO Dengue guidelines in order to standardize the admission criteria and time across hospitals.

## Author Summary

A variety of factors affect the time of admission of Dengue fever cases. These must be investigated, as delayed treatment of this disease can result in death. The authors of this study determined the factors associated with the time of admission among notified Dengue cases of Region VIII, Philippines, from 2008 to 2014. The factors assessed were age and sex of the patient, hospital sector, hospital level, disease severity and the presence of Dengue epidemic. A secondary surveillance data of Dengue was used. The associations were determined using chi-square test and regression. Late admission was more likely amongst adults, severe cases of the disease, public hospitals, tertiary level hospitals, and during the non-epidemic period. In comparison, early admission was more likely in cases concerning children, mild cases of the disease, private hospitals, non-tertiary hospitals and during an epidemic period. Case fatality was significantly associated to children, severe diseases, public hospitals and tertiary hospitals when admitted late. The routine early admission of children should be promoted, as severe cases of Dengue fever are more likely among children. Consistent admission criteria for Dengue should be implemented across all hospital sectors and levels.

## Introduction

Dengue fever is endemic in the Philippines. The Department of Health (DOH) reported 59,943 cases between January and September 2014, with an incidence of 60 cases per 100,000 [1]. Sixty five percent of the cases in the country were admitted, while 35% were treated as outpatient [2].

Mortality in Dengue cases is often associated with treatment delay [3]. The delay arises mainly from late hospital admission. Multiple factors influence the time of admission–for example, distance from hospitals or poor recognition of warning signs and symptoms [4]. The ownership of hospitals (ie. whether they are private or public) also has a significant influence on health-seeking behaviour [5, 6]. In private hospitals, early consultations are deterred by the immediate costs [5]. Data suggests that late admissions in public hospitals could be related to the lack of confidence in their services [6]. The presence of an epidemic can also cause a change in the time of admission. In a 2007/2008 epidemic in Rio de Janiero, Dengue cases frequently received primary medical care on the third day from the onset of illness [7]. This study assessed the effect of the 2010 epidemic in Region VIII [8], Philippines on the time of admission.

Differences in the admission time have also been associated with disease severity. Non-severe cases were usually admitted on the third and fourth day since the onset of the disease, while severe cases were admitted later [7]. In a 2006 study by Tomaschek in Puerto Rico, Dengue cases were seen by a clinician at least once, without appropriate assessment of the severe warning signs, before being admitted [9]. Late admission of severe cases among adults has been reported by their unusual clinical presentation, such as severe Dengue with no fever [7].

Early and improved management can reduce morbidity and mortality among Dengue patients (3, 4). In general, case fatality rate (CFR) of 1 may be considered a consequence of insufficient management, late diagnosis and hospital admission [10]. Therefore, understanding the factors related to the time of hospitalization can improve Dengue management.

In this study, we determined the factors associated with hospital admission time among Dengue cases. The study period was from 2008 to 2014 in Region VIII, Philippines. The results of this study may provide recommendations for organizational policies and treatment protocols to improve the admission time of Dengue patients.

## Methods

Dengue cases are reported weekly to the Philippines Integrated Disease Surveillance and Response (PIDSR) through passive surveillance and active sentinel surveillance [11]. We conducted an exhaustive retrospective sampling and analysis of the notified cases of Region VIII. The study period lasted from January 1, 2008 to December 31, 2014.

The case definition of Dengue was based on the 1997 World Health Organization (WHO) classification [11]. For this study, Dengue fever (DF) was operationally defined as a mild case. The Dengue hemorrhagic fever (DHF) and Dengue shock syndrome (DSS) were grouped together, and defined as a severe case.

We measured the outcome variable as the time of admission, measured in the number of days between the onset of illness and the time of admission. The variable was categorized into early (0–2 days), regular (3–5 days) and late (6 or more days) admission. These categories were arbitrarily derived from the clinical phases and period of Dengue: febrile, critical and recovery [12].

We restricted the explanatory variables to the set of data available in the PIDSR, namely disease severity, age and sex of the patient, hospital sector, hospital level and period of admission. The hospital sector was categorized into public and private ownership. The hospital level was categorized into tertiary and non-tertiary hospitals [13]. The tertiary hospitals were generally located in the urban area of the region. The period of admission was divided into the epidemic (2010) and non-epidemic period (2008, 2009, 2011, 2012, 2013, 2014). The year 2010 was defined as the epidemic period because of the remarkable increase in the number of cases compared with other years.

Only admitted cases were included in the study. Values with a missing date of admission and onset of illness were excluded. Cases with an admission time of over 90 days were excluded, since Dengue can only be confirmed serologically by IgM until 90 days [14].

We established the association of the explanatory variables to the outcome variable through chi-square test in the univariate analysis and ordinal logistic regression in the multivariable analysis. We also compared the case fatality rates (CFR) across varying times of admission by different factors using linear regression. The p-value was set at <0.05.

The model used was the proportional odds assumption for ordinal logistic regression [15]. It assumed that the coefficients between outcome categories (time of admission) were similar. We assumed the coefficients of early admission versus regular and late admission were similar, in regular admission versus late admission. This is called the proportional odds assumption or the parallel regression assumption. All statistical analyses were performed with RStudio Version 0.98.1103 and packages MASS, Hmisc, reshape2, foreign, ggplot2, rms, gridExtra (The R Foundation for Statistical Computing, Vienna, Austria; http://www.r-project.org).

The study was authorized and exempted from the bioethics approval though the DOH Region VIII director. The probability of physical, psychological, social, or economic harm occurring as a result of being included in the research study was minimal. The names of the patients were replaced with unique keys, ensuring the confidentiality of the information gathered.

## Results

### A. General description of the Dengue fever cases

**Fig 1:**
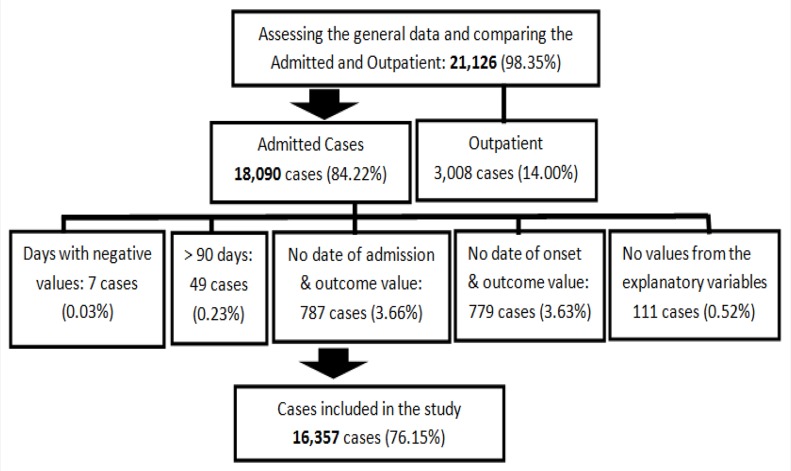
Dengue notified cases of Region VIII, Philippines 2008–2014 included in the study.

There were 21,480 reported cases from 2008 to 2014 in Region VIII. The epidemic period (2010) accounted for 11,974 cases (56.68%). Only 1.40% of these cases were confirmed with laboratory tests. Most cases (85.74%) were admitted in hospitals. A total of 16,357 admitted cases (76.15%) were included in the study after excluding the repeated cases (1.38%), non-Region VIII cases (0.27%), outpatients (14.00%), missing values of the outcome variable (7.29%), cases with missing values of the outcome variable entries (1.38%), and no values for the explanatory variable (0.52%), (Fig 1).

Males and females were equally distributed among admitted cases. Most of the admitted cases were children (70.09%), and those suffering from a mild disease (64.00%). Most of the cases were reported from the public sector (69.82%) and non-tertiary hospitals (62.76%). The number of cases was equally proportional during the epidemic and non-epidemic period (Table 1).

**Table 1:** Descriptive characteristics of the admitted Dengue cases of Region VIII Philippines, 2008–2014.

Characteristics	Frequency	Percentage
**Sex**		
Female	7,845	47.96%
Male	8,512	52.04%
**Age**		
0–14 years old	11,464	70.09%
15–64 years old	4,829	29.52%
>65 years old	64	0.39%
**Severity**		
Mild	10,468	64.00%
Severe	5,889	36.00%
**Sector**		
Public	11,420	69.82%
Private	4,937	30.18%
**Level**		
Tertiary	6,091	37.24%
Non-tertiary	10,266	62.76%
**Period**		
2010 Epidemic	7,963	48.68%
Nonepidemic	8,394	51.32%
**Total**	**16,357**	100%

### B. Case distribution related to time of admission

The univariate analysis (Table 2) indicated significant association of the time of admission to disease severity, patient’s age, hospital sector, hospital level and period of admission (p<0.05). There was no association between sex and time of admission.

**Table 2:** Factors associated to the time of admission of Dengue cases in Region VIII Philippines from 2008–2014.

	Time of Admission						
Factors	0–2 days		3–5 days		> = 6 days		p-value
	(early)		(regular)		(late)		
	N	%	n	%	N	%	
**Severity**							
Mild	2,582	24.67	6,768	64.65	1,118	10.68	<0.05
Severe	1,159	19.68	4,048	68.74	682	11.58	
**Age**							
0–14 years old	2,796	24.39	7,509	65.50	1,159	10.11	<0.05
15–64 years old	928	19.22	3273	67.78	628	13.00	
> = 65 years old	17	26.56	34	53.12	13	20.31	
**Sex**							
Female	1,787	22.78	5,233	66.70	825	10.52	0.13
Male	1,954	22.96	5,583	65.59	975	11.45	
**Sector**							
Public	2,466	21.59	7,646	66.95	1,308	11.45	<0.05
Private	1,275	25.83	3170	64.21	492	9.97	
**Level**							
Tertiary	1,128	18.52	4,287	70.38	676	11.10	<0.05
Non-tertiary	2,613	25.45	6,529	63.60	1124	10.95	
**Period**							
Epidemic	1925	24.17	5234	65.73	804	10.10	<0.05
Non-epidemic	1816	21.63	5582	66.50	996	11.87	

Severe cases were admitted later than mild ones, and adult cases were admitted later than child cases. Although elderly cases (aged 65 and above) were also admitted later than child cases, there were only 64 elderly cases in this study.

Public hospital cases were admitted later than private hospital cases. Similarly, tertiary hospital cases were admitted later than non-tertiary ones. Cases during the non-epidemic period were admitted later than cases during the 2010 epidemic.

### C. Time of admission by disease severity among different factors

We stratified and described the time of admission by disease severity for sex and age of the patient, hospital level, hospital sector and period of admission (S1 Table). Both mild cases and severe cases, indicated significant association of the time of admission with age, hospital sector, hospital level and period of admission (p<0.05) but not with sex. Mild cases comprised 69% of the cases in public hospitals, as opposed to 52% in private hospitals. Mild cases made up 78% of the cases in non-tertiary hospitals, as compared with 40% in tertiary hospitals. Both mild and severe cases from public and tertiary hospitals were admitted later than those in private and non-tertiary hospitals.

Both severe and mild cases have a significant association between the time of admission and age (p<0.05). Severe cases made up 37.86% of cases among children, as opposed to 31.62% in adults. There were only 22 severe cases among the elderly. Adults were admitted later than children. The time of admission for both severe and mild cases was also significantly associated to the epidemic or non-epidemic period.

### D. Time of admission by age, sex, facility type, disease severity and epidemic period

We found significant association between disease severity, age of patient, hospital sector, hospital level and period of admission in our multivariate analysis (Table 3).

**Table 3:** Ordinal Logistic Regression of the factors associated to the time of admission of Dengue cases in Region VIII Philippines from 2008 to 2014.

	Coefficient	Standard Error	
			P value
		SE	
**Severity**			
Reference: Mild			
Severe	0.17	0.04	<0.05
**Age**			
Reference: 0–14 years old			
15–64 years old (adults)	0.32	0.04	<0.05
> = 65 years old (elderly)	0.41	0.28	1.37
**Sex**			
Reference: Female			
Male	0.02	0.03	0.62
**Hospital Sector**			
Reference: Private			
Public	0.34	0.04	<0.05
**Hospital Level**			
Reference: non-tertiary			
Tertiary	0.29	0.04	<0.05
**Period of Admission**			
Reference: Epidemic			
Non-epidemic	0.14	0.03	<0.05

Late admission was more likely when the following factors occurred: a) severe case of the disease (p<0.05) in comparison with the mild type; b) adult patients rather child patients (p<0.05); c) public hospital admission as opposed to private hospital (p<0.05); d) tertiary level hospital admission rather than non-tertiary (p<0.05); and e) non-epidemic period as opposed to an epidemic period (p<0.05). The factor with the highest influence was public sector hospital, with a higher probability of late admission in comparison with the private sector.

There was no association between sex and the time of admission (p = 0.62). There was no significant difference in admission time between the elderly and children (p = 1.37).

The results obtained in S3 Table were used to assess the proportional odds assumption of the model. The differences in the predicted coefficients in each level were only within the range of 0.01 to 0.4. This suggests that the coefficients at different levels of the time of admission were similar in all the risk factors assessed, and that the proportional odds assumption was held in the model (15).

### E. Case fatality rate and time of admission

**Fig 2:**
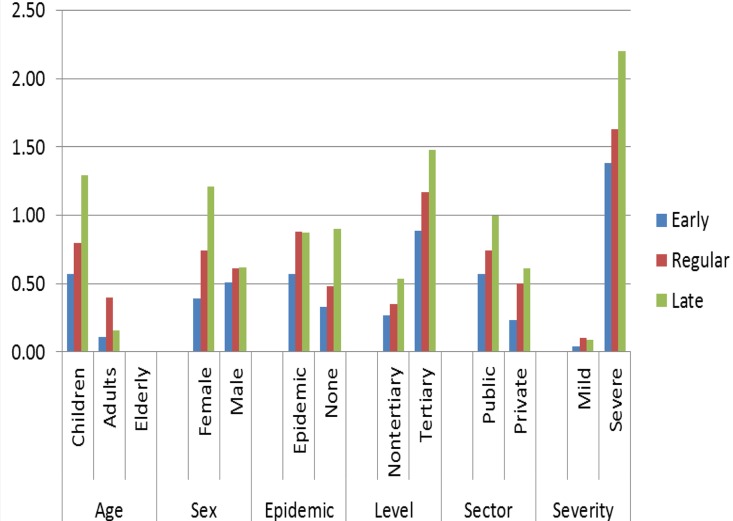
Case fatality rate in different times of admission by age, sex, epidemic period, hospital level, hospital sector and disease severity.

There were 106 deaths among the notified dengue cases with an overall CFR of 0.65. There was a general increase in the CFR across time of admission. The CFR was equal or greater than 1 during late admission amongst children, severe disease, tertiary hospital, public hospital and females (Fig 2). However, the CFR at different times of admission was only significantly associated with age, severity, hospital level and hospital sector (p<0.05). It was not associated with sex (p = 0.32) and epidemic period (p = 0.15) (S2 Table).

## Discussion

Between 2008 and 2014, most Dengue cases in Region VIII in the Philipppines involved children, mild cases of the disease, public sector and non-tertiary hospitals. Cases from the epidemic period comprised almost half of the admitted cases.

The predominance of Dengue fever amongst children in this study supports the 2002 study by Guzman, describing Dengue fever as a disease predominantly affecting children [16]. Nonetheless, certain studies have reported a changing epidemiologic distribution in age groups [10].

The predominance of admitted cases among all notified cases in this period can be attributed to the large number of hospitals included in the surveillance system as reporting units. Hospitals usually take the reports of inpatients rather than the outpatients. In 2015, Edillo estimated that 65% of the Dengue cases in the Philippines concern inpatients [2]. In Region VIII, the public sector and non-tertiary hospitals collectively have the largest number of beds [17], which suggests a reporting bias in favour of these hospitals, as opposed to private and tertiary hospitals.

The majority of cases were admitted during the period between the third and fifth day from the onset of the disease. Cases admitted later than five days were least common. These findings correspond to a similar study, conducted by Velasco in 2014, which found a mean value of 4 days of illness prior to admission for children and adults [18]. According to a 2013 study by Moraes, there is a higher probability of death among patients who had their case notified after four days from the onset of symptoms compared with those whose cases were notified earlier [19].

The critical phase of the Dengue fever ranges from the third to fifth day, during which time patients experience weakness, abdominal pain, vomiting, plasma leakage, bleeding or hypotension [20]. This is the period when they are most likely to seek hospital admission [7]. The overall CFR of 0.65 for Region VIII was consistent with the national average [1,8]. The CFR for Region VIII ranged from 0.50 to 1.00 per 100 cases [1,8].

The five main results corresponding to the significant factors associated to the time of admission were:

### A. Lateness and severity

Severe cases were more likely to be admitted late in comparison to mild ones. The CFR of severe cases exceeded 2 during late admission. Patients with mild disease generally seek medical attention during the period 2–4 days after the onset of illness [14,21]. Under the 1997 classification, severe cases of Dengue are categorised as cases where hemorrhage or shock occurs [3, 12]. These symptoms are generally present later than two days from the onset of illness, or at a late stage [14, 16, 18] which falls on the period of late admission. Hence, late admission may have been favoured among severe cases with high CFR based on the 1997 classification. In 2009, WHO reclassified Dengue fever based on early warning signs to detect early on cases likely to deteriorate [22]. This new classification has increased sensitivity to severe cases of Dengue [23]. However, its use in the Region VIII surveillance was only initiated in 2013, therefore it was not used in this study.

Dengue infection severity can vary among individuals. It has been suggested that there are higher levels of viral load in severe cases correlating to the seriousnes of the disease [24]. The mechanism remains to be determined through viral serotyping, investigating susceptibility and tracing the sequence of the infection [25].

### B. Lateness and age

The symptoms of Dengue seemed to cause little alarm for adult patients, who were generally more likely to experience late admission than children (Table 2). On the other hand admission time amongst the elderly were similar to those found amongst children.

A study in Southeast Asia found that cases of Dengue amongst adults showed mild signs and symptoms [25]. In contrast, a majority of the severe cases occur in children aged 2–15 years. Adults apparently acquire immunity from primary infection and avoid DHF [25]. S1 Table of this study is consistent with literature revealing a higher percentage of severe cases in children rather than adults [16, 25]. Furthermore, the CFR among children during late admission exceeded 1%. This emphasizes the important practice of early admission among children who are likely to have severe Dengue.

### C. Lateness and hospital sector

Dengue patients treated in public hospitals were more likely to be admitted late than those in private hospitals, particularly in mild cases. There was no difference in admission time between private and public sectors in both mild and severe cases (S1 Table). The difference in admission time beween the hospital sectors suggests that there may be a disparity in the clinical practice between public and private hospitals. The CFR in public hospital during late admission approached 1% in comparison to the private hospital.

The financial capacity and remote location of patients influence their health-seeking behaviour. Public hospitals are frequently visited by patients with more limited economic resources who may endure symptoms of disease until they are critically ill [26]. Three quarters of the patient load in our study were admitted by public hospitals. In these hospitals, the doctor’s fee and accommodation are government-subsidized [27]. However, public hospitals are often so full that there are no vacant beds to accommodate additional admissions. They also have meager medical supplies and insufficient personnel, which can lead to long waiting hours [27]. As a result of poor health facilities and the patients’ limited financial capacity, the late admission is more likely in public hospitals than private ones. In contrast, private hospitals experience a more reduced occupancy rate [27]. According to anecdotal descriptions from Region VII, private hospitals tend to have higher and faster admission rates, even in mild cases, in order to increase the number of clients for commercial purposes.

### D. Lateness and hospital level

In a 2013 report by the National Statistics Coordinating Board, 3 public hospitals and 7 private hospitals in Region VII were classified as tertiary hospitals, while 46 public hospitals and 21 private hospitals were classified as non-tertiary hospitals [28].

Most of the cases in this study were reported from non-tertiary hospitals (63%). In general, tertiary hospitals admit Dengue cases later than non-tertiary ones (Table 3). When we stratified the time of admission by disease severity, it appeared that late admission among tertiary hospitals was evident in both mild and severe cases (S1 Table). There may be a disparity in the time of admission for mild cases.

Most non-tertiary hospitals are funded by the private sector or local government. Non-tertiary hospitals are generally perceived as providing low quality services because of their meager resources [27]. Patients who bypass their services and seek treatment in better equipped tertiary hospitals have to travel to the urban area, where national public hospitals or large private hospitals are situated [27]. These tertiary hospitals have a higher likelihood of late admission than non-tertiary hospitals. The CFR in tertiary hospitals during late admission exceeded 1. Tertiary hospitals are overburdened, because Dengue cases are more common in densely populated urban areas where vectors proliferate [10].

### E. Lateness and epidemic

The 2010 epidemic accounted for the highest number of reported Dengue cases. The likelihood of late admission was higher during the non-epidemic period than the epidemic period. This is contrary to evidence suggesting that a high volume of patients can overburden and delay health services [29, 30].

During the 2010 epidemic, the number of admissions increased to five times the average per year as compared to the non-epidemic period. The non-epidemic period covered 6 years which averaged 1,399 cases per year. The 2010 epidemic had 7,963 cases in a single year (Table 1). Anecdotal reports from the hospital staff revealed the use of the lobby and room extensions to accommodate the large number of patients.

During the 2010 epidemic, public campaigns on early consultation were conducted. Individuals with fever lasting two or more days were advised to immediately seek consultation [8]. The community became more prone to suspecting any febrile condition to be Dengue fever. Anecdotal observations also suggest that increased awareness encouraged to promptly diagnose and admit Dengue cases. This may explain the lower likelihood of late admission during the epidemic period. However, the earlier admission may have occurred at the expense of the quality of services.

### Limitations

The 1997 WHO Dengue guidelines classification was used in this study. The 1997 and 2009 Dengue guidelines use different criteria for categorizing clinical Dengue case severity [3, 9]. The 1997 guidelines favour late admission for severe cases, since haemorrhagic and shock presentations naturally occur during the late phase of the disease [3]. The 2009 guidelines screen through warning signs that could occur during the early to late phase of the disease. However, the 2009 WHO classification was only adopted in Region VIII surveillance data in 2013. As a result, our study only included a limited number of cases categorized under this new classification.

The majority of Dengue cases were suspected and not laboratory confirmed, and thus based on clinical presentation. Due to the lack of serological laboratory confirmation, it is possible that cases that were not caused by Dengue were nonetheless included in the study. Other febrile illnesses may have been classified as Dengue in the study, especially during the 2010 epidemic. It is also worth bearing in mind that other diseases may have been classified as Dengue for insurance coverage reasons, seeing as a higher reimbursement is granted for cases of Dengue fever. An overestimation of the number of cases may have occurred, but there is no evidence that this affected the distribution of the cases in terms of admission time.

Factors such as chronic diseases, concomitant diseases, immunologic status, Dengue virus serotype, secondary infection, body mass index, inter-hospital transfer, hospital accessibility, or socioeconomic level may also have influenced the time of admission. However, these factors were not included in the analysis as they are not part of the surveillance information collected.

### Conclusions and recommendations

In Region VIII, the Philippines, late admission of Dengue cases was more likely among adults, public hospitals, tertiary hospitals and during non-epidemic period. Among the factors, the highest likelihood of late admission was in public hospitals than in private hospitals.

Late admission was associated with hospital sector and level. These may be influenced by patients’ financial capacity, a high patient load and lack of resources in healthcare facilities, the geographic location of hospitals, and noncompliance with Dengue guidelines.

The study also suggests a higher likelihood of early admission among children in comparison to adults. This behavior should be encouraged, as severe cases are more common among children than adults. The severe cases of Dengue were more likely to be admitted late than mild cases. However, severe diseases have late presentation which may have favoured their late admission.

A CFR of 1.00 or greater was observed during the late admission of children, severe diseases, tertiary hospitals and public hospitals. These suggest that admission time and management should be improved in these factors to minimize death.

Earlier admission was more likely during the 2010 Dengue epidemic in Region VIII than during the non-epidemic period, suggesting behavioural change attributable to increased awareness of the disease. Improvements in behaviour related to admission time can be brought about through increased knowledge, practice and compliance to the Dengue guidelines by the healthcare workers and population.

We recommend the improvement and consistent implementation of hospital admission practices. Firstly, in order to facilitate early admission and prevent fatality, health care workers should be able to identify severe Dengue fever cases. The identification of severe cases may improve with the use of the 2009 WHO Dengue guidelines classification, which examines warning signs and indicates when the patient is admissible. Trainings should be conducted among healthcare professionals. The use of the 2009 WHO guidelines as the basis for admission, and the confirmation of the diagnosis through serological tests should be discussed and agreed on among doctors, surveillance staff and health insurance personnel. The guidelines are expected to standardize the diagnosis and hospital admission time across different hospital sectors and levels.

Through public campaigns, the practice of early admission among children suffering from severe cases of the Dengue disease should be promoted among healthcare professionals. During epidemics, the provision of examining stations exclusively for children can ensure the on-going practice of early hospitalization.

Secondly, the reasons for late admission in public and tertiary hospitals must be studied further in order to improve promptness in Dengue case management. Hence, further research will be needed to assess how admission time is affected by the volume of cases, accessibility and out-of-pocket expense of these hospitals The quality of health care services must also be further explored to understand how the health system adapted during the epidemic, and why admission time was earlier during the epidemic rather than non-epidemic period.

## Supporting Information

S1 Table Time of admission by disease severity among different factors (PDF)

S2 Table Case fatality rate among factors in different times of admission (PDF)

S3 Table Table for testing the proportional odds assumption (PDF)
