# Supplementary material for: Factors Associated with the Time of Admission among Notified Dengue Fever Cases in Region VIII Philippines from 2008 to 2014
Source: PLoS Negl Trop Dis. 2016 Oct 25;10(10):e0005050. doi: 10.1371/journal.pntd.0005050 (PMC5079576; doi:10.1371/journal.pntd.0005050)
Supplement: S1 Table — (PDF) [file pntd.0005050.s001.pdf]

S1 Table: Time of admission by disease severity among different factors

	MILD							SEVERE						
	**Early**	**%**	**Regular**	**%**	**Late**	**%**	**p-value**	**Early**	**%**	**Regular**	**%**	**Late**	**%**	**p-value**
**Sex**														
Female	1235	24.80	3244	65.14	501	10.06	0.15	552	19.27	1989	69.42	324	11.31	0.54
Male	1347	24.54	3524	64.21	617	11.24		607	20.07	2059	68.09	358	11.84	
**Age**														
Children	1895	26.60	4536	63.67	693	9.73	<0.05	901	20.76	2973	68.50	466	10.74	<0.05
Adults	678	20.53	2208	66.87	416	12.60		250	16.37	1065	69.74	212	13.88	
Elderly	9	21.43	24	57.14	9	21.43		8	36.36	10	45.45	4	18.18	
**Sector**														
Public	1890	23.99	5111	64.89	876	11.12	<0.05	576	16.26	2535	71.55	432	12.19	<0.05
Private	692	26.71	1657	63.95	242	9.34		583	24.85	1513	64.49	250	10.66	
**Level**														
Non-tertiary	2087	25.97	5112	63.61	838	10.43	<0.05	526	23.60	1417	63.57	286	12.83	<0.05
Tertiary	495	20.36	1656	68.12	280	11.52		633	17.30	2631	71.89	396	10.82	
**Period**														
Epidemic	1641	26.49	3936	63.55	617	9.96	<0.05	640	18.41	2447	70.38	390	11.22	<0.05
None	941	22.02	2832	66.26	501	11.72		519	21.52	1601	66.38	292	12.11	
