# Supplementary material for: Factors Associated with the Time of Admission among Notified Dengue Fever Cases in Region VIII Philippines from 2008 to 2014
Source: PLoS Negl Trop Dis. 2016 Oct 25;10(10):e0005050. doi: 10.1371/journal.pntd.0005050 (PMC5079576; doi:10.1371/journal.pntd.0005050)
Supplement: S2 Table — (PDF) [file pntd.0005050.s002.pdf]

S2 Table: Case fatality rate among factors in different times of admission

Factor	Category	Early	Regular	Late	p-value
**Age**	Children	0.57	0.80	1.29	<0.05
	Adults	0.11	0.40	0.16	
	Elderly	0.00	0.00	0.00	
**Sex**	Female	0.39	0.75	1.21	0.32
	Male	0.51	0.61	0.62	
**Epidemic**	Epidemic	0.57	0.88	0.87	0.15
	None	0.33	0.48	0.90	
**Level**	Nontertiary	0.27	0.35	0.53	<0.05
	Tertiary	0.89	1.17	1.48	
**Sector**	Public	0.57	0.75	0.99	<0.05
	Private	0.24	0.50	0.61	
**Severity**	Mild	0.04	0.10	0.09	<0.05
	Severe	1.38	1.63	2.20	
