# Supplementary material for: Factors Associated with the Time of Admission among Notified Dengue Fever Cases in Region VIII Philippines from 2008 to 2014
Source: PLoS Negl Trop Dis. 2016 Oct 25;10(10):e0005050. doi: 10.1371/journal.pntd.0005050 (PMC5079576; doi:10.1371/journal.pntd.0005050)
Supplement: S3 Table — (PDF) [file pntd.0005050.s003.pdf]

S3 Table: Table for testing the proportional odds assumption

Factors	Categories	N	Y >=1	Y>=2	Y>=3
**Severity**	Mild	10468	1.116525	1.116525	1.116525
	Severe	5889	1.406368	1.406368	1.406368
**Age**	0-14year/s old	11464	1.131448	1.131448	1.131448
	15-64years old	4829	1.435956	1.435956	1.435956
	65=>years old	64	1.016934	1.016934	1.016934
**Sex**	Females	7845	1.220841	1.220841	1.220841
	Males	8512	1.210807	1.210807	1.210807
**Sector**	Private	4937	1.055063	1.055063	1.055063
	Public	11420	1.289503	1.289503	1.289503
**Level**	Non-tertiary	10266	1.074599	1.074599	1.074599
	Tertiary	6091	1.481564	1.481564	1.481564
**Epidemic**	Epidemic	9671	1.175514	1.175514	1.175514
	None	6686	1.275210	1.275210	1.275210
**Overall**		16357	1.215613	1.215613	1.215613
